# A Novel Mechanism for Small Heat Shock Proteins to Function as Molecular Chaperones

**DOI:** 10.1038/srep08811

**Published:** 2015-03-06

**Authors:** Kaiming Zhang, Anastasia N. Ezemaduka, Zhao Wang, Hongli Hu, Xiaodong Shi, Chuang Liu, Xinping Lu, Xinmiao Fu, Zengyi Chang, Chang-Cheng Yin

**Affiliations:** 1 Department of Biophysics, College of Basic Medical Sciences, Peking University Health Science Centre, Beijing 100191, China; 2 State Key Laboratory of Protein and Plant Gene Research, School of Life Sciences, Peking University, Beijing 100871, China; 3 School of Life Sciences, Tsinghua University, Beijing 100084, China; 4 Centre for Protein Science, Peking University, Beijing. 100871, China

## Abstract

Small heat shock proteins (sHSPs) are molecular chaperones ubiquitously present in all forms of life, but their function mechanisms remain controversial. Here we show by cryo-electron microscopy and single particle 3D reconstruction that, at the low temperatures (4–25°C), CeHSP17 (a sHSP from *Caenorhabditis elegans*) exists as a 24-subunit spherical oligomer with tetrahedral symmetry. Our studies demonstrate that CeHSP17 forms large sheet-like super-molecular assemblies (SMAs) at the high temperatures (45–60°C), and such SMAs are apparently the form that exhibits chaperone-like activity. Our findings suggest a novel molecular mechanism for sHSPs to function as molecular chaperones.

As a family of molecular chaperones, small heat shock proteins (sHSPs) are characterized by having a low subunit molecular mass of 12–43 kDa, possession of a conserved α-crystallin domain^1^,^2^,^3^,^4^ and formation of large oligomers^5^,^6^,^7^. They are known to act as ATP-independent “holdase” molecular chaperones that bind to the non-native substrate proteins and prevent them from forming irreversible aggregations, under stress conditions^8^,^9^; The client proteins would then be released for refolding with the help of other ATP-dependent chaperones when the conditions become optimal^10^,^11^,^12^,^13^,^14^.

The sHSPs are ubiquitously present in all three kingdoms of organisms^1^,^3^. The number of genes encoding sHSPs in different organisms varies greatly, from as few as one to as many as nineteen^4^. Investigations of sHSPs from mammals, yeast, plants and bacteria have unveiled their role in thermotolerance such that the over-expression of eukaryotic recombinant sHSPs could increase the thermotolerance of *E. coli* cells^15^,^16^,^17^. The sHSPs have been observed to exist as large homo-oligomers and the reversible dissociation of their oligomers has been reported to be important for their enhanced chaperone activity at high temperatures^7^,^18^,^19^,^20^,^21^,^22^,^23^. Such oligomeric dissociation presumably increases the exposure of their hydrophobic regions, which are buried in the oligomers at the normal physiological conditions but needed for binding non-native substrate proteins^6^,^7^,^19^,^24^.

Among the fourteen sHSPs present in *Caenorhabditis elegans*^25^, the structure and function of the CeHSP17 protein (with the gene named as F52E1.7 on the website www.wormbase.org) is the least understood. Recently, we reported that the CeHSP17 protein enables *Escherichia coli* cells to grow at a lethal temperature of 50°C^26^, the mechanism of which remains to be defined. In an attempt to elucidate the action mechanism of the CeHSP17 protein, here we determined its structure at both low and high temperatures using electron microscopy. Our studies demonstrate that the CeHSP17 protein forms homogenous spherical oligomers at low temperatures, but transforms into sheet-like super-molecular assemblies at high temperatures. Intriguingly, we found that only the super-molecular assemblies but not the spherical oligomers of the CeHSP17 protein most likely exhibit chaperone-like activity in preventing the stress-induced aggregation of model substrate proteins. Our findings unveiled a new structural form of sHSPs and shed light onto a novel molecular mechanism for sHSPs to function as molecular chaperones.

## Results

### Heterologous expression of the CeHSP17 protein confers thermotolerance on *E. coli* cells

**Figure 1:**
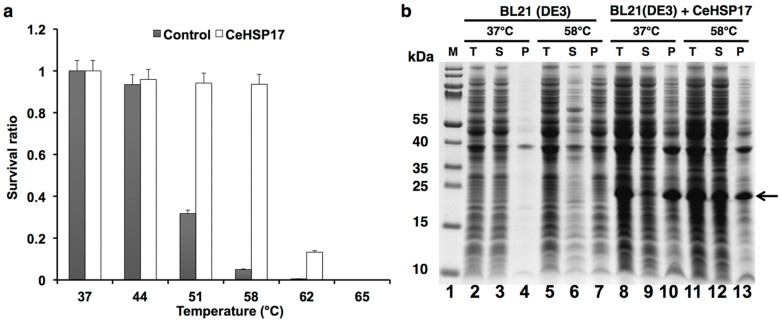
Heterologous expression of CeHSP17 confers thermotolerance on *E. coli* cells and prevents protein aggregation at the heat shock temperatures. (a) The survival ratio of BL21(DE3) cells over-expressing CeHSP17 after being heat shocked at the indicated temperatures. The error bars represent the standard deviation (SD) of three independent experiments. (b) SDS-PAGE analysis results of the proteins present in the total lysates (T), soluble (S) and insoluble pellet (P) fractions of the control cells (i.e., transformed with the empty vector) and the CeHSP17-expressing BL21(DE3) cells heat shocked at 58°C for half an hour or without heat shock treatment (i.e., maintained at 37°C). The protein bands were visualised by Coomassie blue staining. Showing on the left is the sizes of the molecular weight markers (lane 1).

The ability of sHSPs to prevent protein aggregation at high temperature thus to confer thermotolerance on cells has been widely reported^5^,^15^,^18^,^27^. In line with our previous observation that the CeHSP17 protein enables the *E. coli* cells to grow at 50°C^26^, here we demonstrated that the heterologously-expressed CeHSP17 protein is able to confer thermotolerance on *E. coli* cells, resulting in a survival rate of about 100% and 10% upon heat shock treatment at 58°C and 62°C respectively, with the control cells being almost completely killed upon such heat shock treatments (Fig. 1a). By contrast, other sHSPs have been reported to only confer partial thermotolerance on *E. coli* cells upon heat shock treatment even at the lower temperature of 50°C^27^,^28^,^29^,^30^.

We then asked whether the sustainability of these cells was in part due to the ability of the CeHSP17 protein to prevent protein aggregation in the *E. coli* cells. The SDS-PAGE analysis result of the soluble and insoluble fractions of the centrifuged total lysates of the CeHSP17-expressing cells that were heat shocked at 58°C revealed a significant increase in the level of soluble proteins, when compared with the control cells without expressing CeHSP17 but treated at the same temperature (Fig. 1b, lanes 12 and 6). It should be noted that our previous microscopy analysis demonstrated that the *E. coli* cells heterologously expressing the CeHSP17 protein retained their normal morphology when growing at 50°C, with their cytoplasmic contents, inner and outer membranes being clearly visible; while the control cells had lost their normal morphology such that their periplasmic space become hardly visible and their cytoplasmic contents largely disappeared with only electron-dense putative protein aggregates visible^26^. These observations demonstrate that the CeHSP17 protein is able to function as a highly efficient molecular chaperone in the *E. coli* cells.

### The CeHSP17 protein exists as homogeneous spherical oligomers at low temperatures

**Figure 2:**
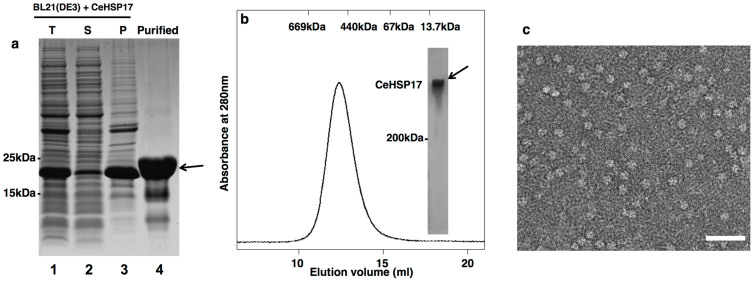
The purified recombinant CeHSP17 protein exists as homogeneous spherical oligomers at low temperatures. (a) SDS-PAGE analysis results of the proteins present in the total lysates (T), soluble (S), insoluble pellet (P) fractions and purified recombinant CeHSP17 protein. (b) Size-exclusion chromatography and non-denaturing pore gradient gel analysis of the purified refolded CeHSP17 protein. (c) Transmission electron microscopy images of the purified refolded CeHSP17 protein. The scale bar represents 50 nm.

The recombinant CeHSP17 protein, being largely found in the insoluble fraction when over-expressed in *E. coli* cells grown at 37°C (Fig. 2a, lane 3), was purified to almost homogeneity under denaturing conditions by nickel-affinity chromatography and refolded via step-wise renaturation against the refolding buffer (buffer A) (Fig. 2a, lane 4). Size exclusion chromatography (Fig. 2b) analysis performed at 4°C demonstrated that the refolded CeHSP17 protein was eluted as a single peak corresponding to an oligomer of ~460 kDa. Non-denaturing pore gradient PAGE analysis performed at 4°C (Fig. 2b, inset) also indicates that the refolded CeHSP17 protein existed as a single high molecular weight oligomer. In line with these observations, our electron microscopy examination of the negatively stained sample, also performed at 4°C, indicated that the refolded CeHSP17 protein exists as uniform spherical oligomers at this temperature, with a diameter of ~13 nm (Fig. 2c). It should be pointed out that the CeHSP17 protein was found to exist as such spherical oligomers at a temperature up to 25°C (data not shown).

### The CeHSP17 protein apparently promotes the aggregation of model substrate proteins at high temperatures

We next investigated whether the CeHSP17 spherical oligomers are able to exhibit chaperone-like activity under *in vitro* conditions. It turned out that no appreciable chaperone-like activity was detected when using reduced insulin as substrates and performing the analysis at 25°C (data not shown). Given that the CeHSP17 protein was previously demonstrated to enable the *E. coli* cells to grow at 50°C^26^, we then performed the chaperone-like activity assay at this temperature. Strikingly, we found that the CeHSP17 protein, instead of suppressing the aggregation of chemically-induced insulin at 50°C, apparently resulted in a significant increase of protein aggregation as indicated by a significant increase of light absorption at 360 nm (Supplementary Fig. S1a). This increase of light absorption was also observed when the CeHSP17 protein was mixed with another model substrate protein, the alcohol dehydrogenase (ADH) and heated at 50°C (Supplementary Fig. S1b).

### The CeHSP17 protein forms super molecular assemblies at high temperatures

To our surprise, the light absorption at 360 nm was found to increase sharply and reach the maximum in less than 2 minutes when the CeHSP17 protein was incubated alone at 50°C (Supplementary Fig. S1c). This is highly comparable to what was observed when the CeHSP17 protein was incubated with the model substrate protein, insulin (Supplementary Fig. S1a) or ADH (Supplementary Fig S1b). In consistent with this, we observed a remarkable reduction of the absorbance peak corresponding to the spherical oligomers when the CeHSP17 protein heated at 50°C was immediately analysed by size-exclusion chromatography (Supplementary Fig. S1d). Such a spherical oligomer absorption peak was clearly observed when the CeHSP17 protein sample was either directly analysed at 4°C, or when the heated CeHSP17 protein was returned to 4°C before subjecting to size-exclusion chromatography analysis (Supplementary Fig. S1d). These observations strongly indicate that the CeHSP17 protein itself is able to self-associate to form large aggregates/assemblies at 50°C. Such large aggregates/assemblies would be ended in the pellet fraction during the pre-centrifugation step and thus became undetectable by size exclusion chromatography analysis.

**Figure 3:**
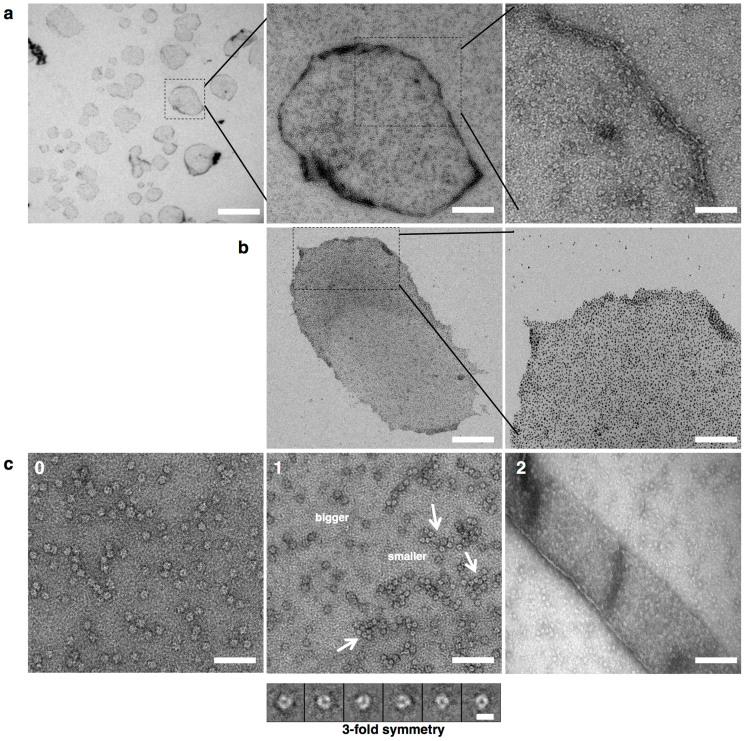
The CeHSP17 protein forms super-molecular assemblies (SMAs) at the high temperatures. (a) Transmission electron microscopy images of negatively stained CeHSP17 protein that was pre-treated at 50°C for 30 min. The scale bars respectively represent 2,000 nm (left panel), 400 nm (middle panel) or 100 nm (right panel). (b) Transmission electron microscopy images of the CeHSP17 proteins labelled with nickel-tethered gold particles after being pre-treated at 50°C for 30 min. The scale bars represent 400 nm (left panel) or 100 nm (right panel). (c) Transmission electron microscopy images of the negatively stained CeHSP17 protein that was untreated (panel c-0) or pre-treated at 50°C for 1 min (panel c-1) or for 5 min (panel c-2). The scale bars respectively represent 100 nm (panel c-0), 100 nm (upper part of panel c-1), 10 nm (lower part of panel c-1) and 200 nm (panel c-2). Arrows in panel c-1 indicate the smaller oligomers (presumably hexamers) that are apparently assembling into the large sheet-like structures.

We next demonstrated by electron microscopy that such aggregates/assemblies formed by the CeHSP17 protein at a high temperature appear as large sheet-like assemblies rather than amorphous aggregates, as judged by their uniform and moderate level of staining (as shown by the results presented in Fig. 3a). To confirm that such sheet-like structures are assemblies of the CeHSP17 protein, we tried to label the histidine tags that were linked to the C-terminus of the CeHSP17 proteins with nickel-tethered gold before applying the samples for electron microscopy analysis. The results presented in Figure 3b clearly demonstrate that such sheet-like structures are assembled from a great number of the CeHSP17 protein units. Notably, such sheet-like assemblies formed upon heating the CeHSP17 protein at a temperature of 45°C or higher. We further performed Fourier transformation analysis to find out if the large sheet-like assemblies are ordered structures. Our analysis indicates that such large sheet-like assemblies contained certain kind of ordered structures only in certain local areas (data not shown). Taken together, our data clearly rules out that such SMAs are amorphous protein aggregates; rather, they are ordered protein assemblies though not as ordered as 2D arrays.

To gain insight into how such sheet-like structures are assembled from the spherical oligomers of the CeHSP17 protein, we applied protein samples from different time points of heating (at 50°C) to electron microscopy analysis. It was observed that, at the initial stage of heating (<1 min), besides the original larger spherical oligomers with a diameter of ~13 nm (Fig. 3c-0 and Fig. 3c-1), smaller transient oligomers emerge with a diameter of ~8 nm (Fig. 3c-1). Such smaller oligomers apparently start to assemble even during the initial stage of heating (as indicated by the arrows in Fig. 3c-1). Upon further incubation, well-formed large sheet-like structures appeared (Figures 3c-2 and 3a). We term such large sheet-like structures super-molecular assemblies (SMAs). The formation of such SMAs are apparently reversible as indicated by the fact that only the small-sized oligomeric forms were detectable (as shown in Supplementary Fig. S1e) when the temperature was lowered from 50°C to 4°C. It should be pointed out that the smaller intermediate oligomers of the CeHSP17 protein, presumably being hexamers as judged from their apparent size in our EM analysis (Fig. 3c-1), were not detected in the size exclusion chromatography analysis as commonly performed at 4°C (Supplementary Fig. S1d). This is most likely due to their reassembly into spherical oligomers upon temperature decrease as the size exclusion chromatography analysis was performed at 4°C, at which the spherical oligomer is the sole assembly form existed (Fig. 3c-0).

### The CeHSP17 super-molecular assemblies exhibit chaperone-like activity

**Figure 4:**
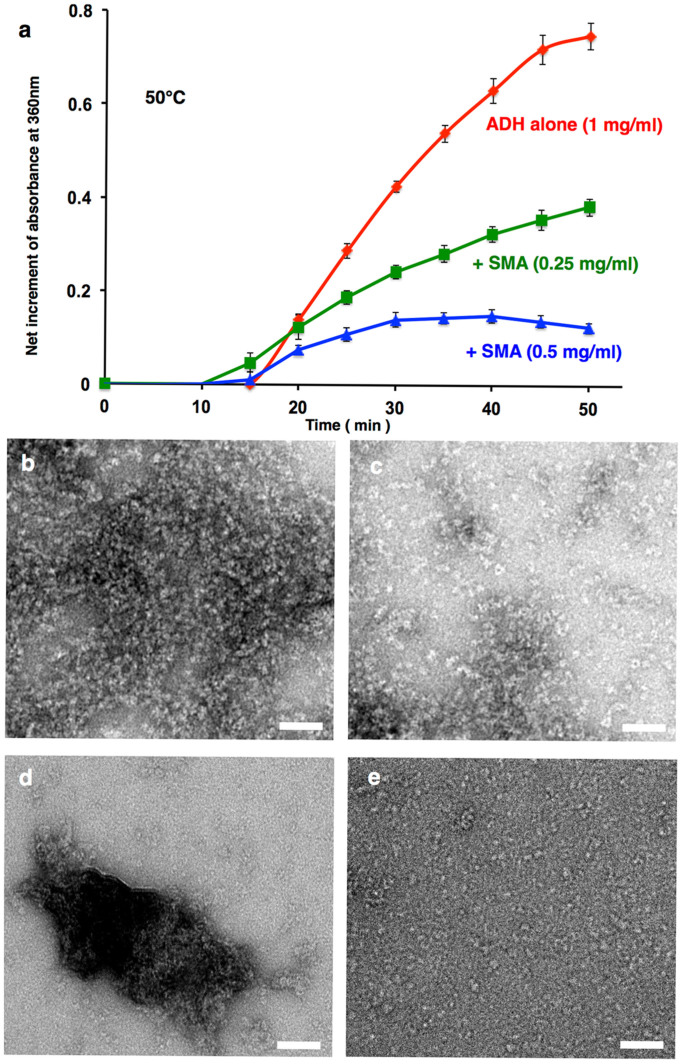
The SMA form of the CeHSP17 protein exhibits chaperone-like activity. (a) Light scattering analysis of the aggregation of ADH induced by heating at 50°C in the absence or presence of the SMAs of the CeHSP17 protein. The light scattering readout at the zero time point was set as zero upon adding the substrate ADH (i.e., the effect of CeHSP17 SMA itself on the light scattering readout was eliminated). The error bars represent the standard deviation (SD) of three independent experiments. (b–e) Transmission electron microscopy images of the following negatively stained samples: The SMAs of the CeHSP17 protein in the presence of denatured ADH (panels b and c), denatured ADH alone (panel d), and untreated ADH alone (panel e). The scale bars represent 50 nm.

We next tried to find out whether these SMAs of the CeHSP17 protein are able to exhibit chaperone-like activity. For this, we first incubated the CeHSP17 protein at 50°C for 30 minutes to pre-form the SMAs before performing the chaperone-like activity assay. Data presented in Fig. 4 indicate that such SMAs are able to act as effective chaperones to suppress the aggregation of unfolded substrate proteins, both the thermally denatured ADH (Fig. 4a) and chemically denatured insulin (Supplementary Fig. S2a), in a dose dependent manner. To verify that it was the SMA forms that exhibited the chaperone-like activity, we repeated the above assay by pre-depleting the SMAs by centrifugation (Our EM analysis indicates that the SMA forms were only detectable in the pellet but not in the supernatant as shown in Supplementary Fig. S2b & S2c). The data demonstrate that the SMA-depleted supernatant fraction is no longer able to suppress the aggregation of the unfolded substrate proteins, either the thermally denatured ADH (Supplementary Fig. S2d) or the chemically denatured insulin (Supplementary Fig. S2e).

We further investigated the status of the SMAs upon incubation with its substrate proteins at the high temperatures. The electron microscopy data indicate that, after binding to the substrates, the original sheet-like structures were no longer observable; instead, a certain kind of large protein structures (Fig. 4b) together with well-separated small protein particles (Fig. 4c) were clearly seen. The appearances of these structures are different from those of the SMAs (Fig. 3a), the CeHSP17 spherical oligomers (Fig. 3c-0, 3c-1), the aggregates formed by denatured ADH (Fig. 4d), or the untreated ADH (Fig. 4e). Conceivably, these large structures (Fig. 4b) represent the ADH-bound SMAs while the small particles (Fig. 4c) represent the CeHSP17-ADH complexes dissociated from the ADH-bound SMAs.

### The cryo-EM structure of the CeHSP17 spherical oligomers

**Figure 5:**
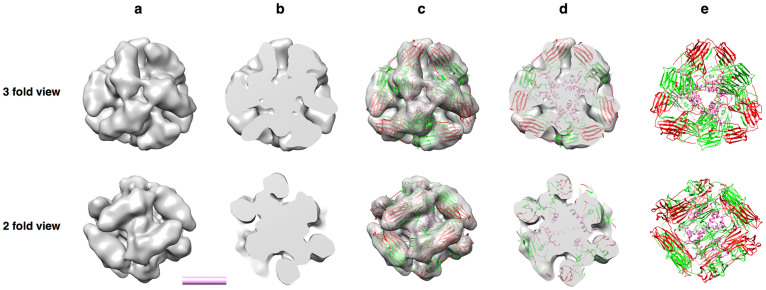
Three-dimensional reconstruction of the CeHSP17 spherical oligomers. The Cryo-EM density maps are viewed as a whole (panel a), as cutting through the centre (panel b), as the crystal structure of wheat HSP16.9 dimer (PDB code 1GME; with the two monomers coloured in red and green respectively) being docked in (panel c), as cutting through the centre of the maps shown in panel c (panel d). Three-dimensional structure model of the CeHSP17 spherical oligomers as obtained from crystal structure docking (panel e). The centre in panel e indicates where the N-terminal segments (pink) of the CeHSP17 protein are located. The EM maps and the 3-D structure model are viewed along the 3-fold and 2-fold symmetry axes. The scale bar represents 5 nm.

To elucidate the structural basis for the transformation from the inactive CeHSP17 spherical oligomer to the active SMAs, we next performed single particle 3D reconstruction of CeHSP17 spherical oligomer from cryo-electron microscopy (cryo-EM) images. For this purpose, we used a total of 14,217 particles collected from 106 cryo-EM images obtained at 4°C and achieved a resolution of 14.5 Å (gold standard criterion, 0.143 Fourier shell correlation, Supplementary Fig. S3). The 3D reconstruction data reveal that the oligomers exist as spherical structure with a diameter of ~130 Å, composing of 12 rod-like “building blocks” with tetrahedral symmetry (Fig. 5a). Unlike the other sHSPs^6^,^7^ these spherical particles apparently are not cage-like, with no hollow space at the centre (Fig. 5b). To explore how the subunits interact with each other in such oligomers, we docked the crystal structure of wheat HSP16.9 dimer (PDB-ID 1GME)^7^, a homologue of CeHSP17, into our cryo-EM density map. As shown in Fig. 5c and Fig. 5d, the crystal structure of the HSP16.9 dimer could be fitted nicely into the 12 rod-like “building blocks”, indicating that the building blocks of the CeHSP17 oligomer are most likely in the forms of dimers. It follows that the CeHSP17 spherical oligomer is composed of 24 subunits, which is consistent with its apparent molecular mass of 460 kDa as determined by size exclusion chromatography (Fig. 2b). Notably, the N-terminal segments, which are believed to bind substrate proteins in other sHSPs^21^,^31^, of all the 24 subunits seem to be buried in the centre of the oligomer and interact with each other (Fig. 5d and Fig. 5e).

## Discussion

**Figure 6:**
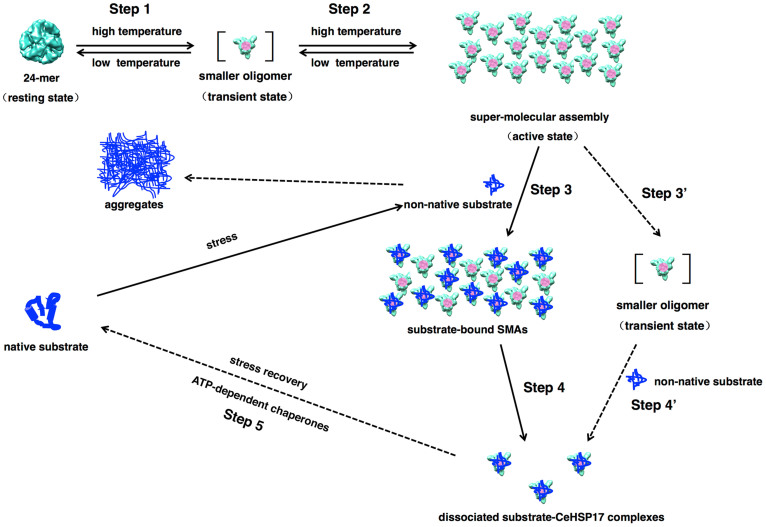
The proposed chaperone mechanism for CeHSP17. The CeHSP17 protein exists as spherical 24-mer (resting state) that lacks chaperone-like activity at the low temperatures. The spherical 24-mer transforms into the unstable (transient) smaller oligomers when exposed to the high temperature stress conditions (Step 1). Such unstable smaller oligomers may immediately self-associate into super-molecular assemblies (SMAs) (Step 2). The exposed N-terminal segments (coloured in pink) in the SMAs then interact with partially unfolded substrate proteins and thus prevent their aggregation (Step 3). Such substrate binding conceivably triggers the dissociation of the small CeHSP17-substrate complexes from the substrate-bound SMAs (Step 4). The substrates in the small complexes are presumably released for refolding with the assistance of ATP-dependent molecular chaperones during stress recovery (Step 5). As a less likely alternative, SMA might function through the transient smaller oligomers (Steps 3′ and 4′).

This study was performed in an attempt to unveil the biological function, structure and mechanism of *C. elegans* small heat shock protein CeHSP17, mainly via biochemical and biophysical approaches. Our major findings include the following. First, heterologous expression of the CeHSP17 protein enabled the *E. coli* cells to effectively resist the severe heat shock treatment at 58°C for half an hour. Second, the CeHSP17 protein exists as homogeneous spherical oligomers at the low temperatures, but forms SMAs at the high temperatures. Third, only the SMA forms exhibit chaperone-like activity in suppressing the aggregation of non-native substrate proteins. In light of these observations, we propose a novel mechanism, as schematically illustrated in Fig. 6, to explain how the CeHSP17 protein is activated for binding non-native substrate proteins, during which the formation of SMAs apparently represents a key step.

The major features of this proposed mechanism are as follows. The CeHSP17 protein exists as 24-subunit spherical oligomers that exhibit no chaperone-like activity at the low temperatures, conceivably due to the inaccessibility of the buried N-terminal segments that most likely act as the major substrate-binding sites as implicated for other sHSPs^21^,^31^,^32^,^33^,^34^,^35^. Upon temperature elevation, the 24-subunit spherical oligomers of the CeHSP17 protein will be transformed transiently into the intermediate smaller oligomers (Step 1 in Fig. 6), which in turn quickly re-associate to form the SMAs (Step 2). It is conceivable that the buried N-terminal segments (coloured in pink in Fig. 6) become accessible in the SMAs, allowing the non-native substrate proteins to be bound and thus their aggregation to be prevented (Step 3). The substrate binding may then trigger the dissociation of a certain form of small CeHSP17-substrate complexes from the substrate-bound SMAs (Step 4). Although our data demonstrate that the formation of the SMA form is a prerequisite for CeHSP17 to exhibit chaperone-like activity, we could not rule out the possibility that the SMA functions through the formation of the transient smaller oligomers (as indicated by Steps 3′ and 4′). The substrates in the small complexes are presumably released for refolding with the assistance of other ATP-dependent molecular chaperones during the stress recovery process (Step 5)^10^,^11^,^12^,^13^,^14^.

Our cryo-EM analyses strongly indicate that the hexamers (trimer of dimers) are the building units dissociable from the 24-subunit spherical oligomers for the CeHSP17 protein. In other words, the packing of the dimers in the spherical 24-subunit oligomers is apparently tight around one type of the 3-fold symmetry axis due to strong interactions, but loose in the other types of symmetry axes due to weak interactions (Fig. 5 and Supplementary movie S1). It follows that these hexamer units can be dissociated from the spherical 24-subunit oligomers upon heating (Step 1 in Fig. 6) and in turn re-assembles into the SMAs (Step 2 in Fig. 6).

The formation of super-molecular assemblies under heat shock conditions might be a common property for sHSPs^1^,^36^. For instance, the mammalian HSP28 was found to form large assemblies with a size greater than 2,000 kDa^37^, the calf alpha-crystallin was observed to form assemblies even with a size larger than 500,000 kDa^38^, and the bacterial IbpA was found to form fibrils at high temperatures under both *in vitro* and *in vivo* conditions^39^. Similarly, we demonstrated that, AgsA (a sHSP from *Salmonella enterica* serovar Typhimurium) is able to form long fibrils, exhibiting chaperone-like activities, at the high temperatures^40^. On the other hand, sHSPs from animals have been commonly detected in stress granules (with a molecular size larger than 2,000 kDa) in heat shock-treated cells^37^,^41^,^42^,^43^. In combination with these previous observations, our work strongly implicate that the super-molecular assemblies most likely represent a common functional form of sHSPs. Additionally, we previously demonstrated that the CeHSP17 protein enables the *E. coli* cells to grow at a temperature as high as 50°C, conceivably by maintaining the integrity of the cell envelope^26^. Our findings reported here would suggest that the over-expressed CeHSP17 protein in the *E. coli* cells function in the form of SMAs to protect the cell envelope for the cells to grow at the high temperature of 50°C. This meanwhile implicates that the CeHSP17 protein may play a similar role to protect the cells in the dauer (dormant) *C. elegans* animals, promoting their survival under such harsh environmental conditions as high temperatures^44^,^45^. Whether such SMAs of sHSPs are indeed formed and functional in living organisms certainly merits further exploration, though technically challenging at the current time.

## Methods

### Thermotolerance and cell viability assay of the CeHSP17- transformed cells

For thermotolerance assay, equal number of *E. coli* BL21 (DE3) cells transformed with the pET21a-CeHSP17 or the control pET21a plasmid (lacking any inserted cDNA sequence) were cultured at 37°C in the LB medium supplemented with 100 μg/ml ampicillin to an OD_600_ of 0.3–0.4 before the induction with isopropyl-β-D-thiolgalactoside (IPTG, at a final concentration of 1 mM) for 1–2 hours. Cells were respectively heat shocked in a temperature-controlled water bath at 44°C, 51°C, 58°C, 62°C and 65°C for half an hour. Serial dilutions of cell cultures were made with the LB medium that was pre-warmed to 37°C and 10 μl of each dilution was then plated in triplicates on the LB plates containing 100 μg/ml of ampicillin, and incubated overnight at 37°C for the cells to grow. The survival ratio was calculated as the ratio of the number of colonies derived from the cells treated at the indicated temperatures and that derived from the non-treated cells.

### Protein solubility analysis of the CeHSP17-transformed *E. coli* cells after heat shock treatment

The effect of heat shock on protein solubility was analysed in *E. coli* cells transformed with pET21a-CeHSP17 or the control pET21a plasmid (lacking any inserted cDNA sequence) according to the method we described earlier^26^. Briefly, cells were first cultured overnight at 37°C in the LB medium containing 100 μg/ml ampicillin before diluted into 20 ml fresh LB medium (containing 100 μg/ml ampicillin) and incubated at 37°C for about 3 hours until an OD_600_ of around 0.4 was reached. The protein expression was induced by the addition of IPTG at a final concentration of 1 mM and the incubation for about another 1 hour at 37°C to an OD_600_ of around 0.5. For heat shock treatment, the cells in the culture medium were placed in a 58°C-incubator for half an hour. Aliquots of the culture were then collected and centrifuged at 5000 rpm for 10 minutes to collect cells. The cell pellets were then washed once with buffer A (20 mM Tris-HCl, pH 8.0) before being sonicated in the same buffer. Cell lysates were then centrifuged at 5000 rpm for 30 minutes before both the supernatants and precipitates were analysed with 10% Tricine SDS-PAGE. The protein bands were visualized by Coomassie Blue staining.

### Expression and purification of the recombinant CeHSP17 protein

The coding sequence for the CeHSP17 protein was inserted into the expression vector pET21a after *Nde*I and *Hin*dIII enzyme digestion. The generated recombinant plasmid pET21a-CeHSP17, which expresses the CeHSP17 protein with a His_6_ tag linked to the C-terminus, was then transformed into the *E. coli* strain BL21 (DE3) cells (Transgene) for protein expression under the induction of 1 mM IPTG for 6–7 hours at 37°C. Cells were harvested by centrifugation at 5000 g for 10 minutes at 4°C. The pellet was re-suspended in buffer B (20 mM Tris-HCl, 50 mM NaCl, 20 mM imidazole, pH 8.0) supplemented with the protease inhibitor phenylmethylsulfonyl fluoride (1 mM), and lysed by sonication. The cell lysates were then centrifuged at 15,000 g for 40 minutes at 4°C. For protein solubility examination, both the supernatant and the pellet were subjected to SDS-PAGE analysis. For protein purification, the pellet fraction containing the CeHSP17 protein was re-suspended with buffer C (20 mM Tris-HCl, 50 mM NaCl, 20 mM imidazole, 8 M urea, pH 8.0) and centrifuged at 30,000 g for 15 minutes. The supernatant containing the CeHSP17 protein was then collected and purified by Ni^2+^-chelate affinity chromatography as described by the manufacturer (Bio-Rad). The eluted fractions were collected and analysed by SDS-PAGE before the eluent containing the CeHSP17 protein was dialyzed against buffer A to remove urea. Protein concentration was measured by the BCA assay (Pierce) according to the manufacturer's instructions.

### Size-exclusion chromatography

Analytical size exclusion chromatography was performed on the ÄKTA FPLC system using a pre-packed Superose 6 10/300 GL column (GE Healthcare). For each analysis, a 100 μl protein sample was loaded onto the column, followed by elution with buffer D (20 mM Tris-HCl, 150 mM NaCl, pH 8.0) at a flow rate of 0.4 ml/min. All the samples were first centrifuged at 15,000 g for 10 minutes before loading the supernatant via injection. All experiments were performed at 4°C.

### Non-denaturing pore gradient polyacrylamide gel electrophoresis

Non-denaturing pore gradient PAGE (3–15%) was performed according to the method previously described^46^. Protein bands were visualized by Coomassie Blue staining.

### *In vitro* chaperone-like activity assay

The chaperone-like activity was measured by monitoring the (tris (2-carboxyethyl) phosphine-induced aggregation of insulin or thermal-induced aggregation of ADH (at 50°C) in the absence and presence of the CeHSP17 protein. Various concentrations of the CeHSP17 protein in buffer D were pre-incubated for 30 minutes to the indicated temperatures. Protein aggregation was monitored by measuring the light absorption at 360 nm. The measurements were performed using a SP-752 spectrophotometer (Shanghai TechCorp).

### Electron microscopy

The negative staining of protein samples was performed as we described earlier^40^. During the EM experiments, the CeHSP17 protein (in an Eppendorf tube) was heated in a water bath of 50°C, before quickly transferred onto the EM grid that was pre-heated on a metal block in the same water bath and then immediately stained. Grids were negatively stained with 2% (w/v) uranyl acetate for 10 s. Electron micrographs were recorded with a JEOL 1400 transmission electron microscope. For cryo-electron microscopy, 2 μl of protein solution (0.4 mg/ml) was applied onto a glow-discharged 200-mesh R1.2/1.3 Quantifoil grid. The grid was blotted and rapidly frozen in liquid ethane using a Vitrobot IV (FEI). Micrographs were recorded on a 4k × 4k CCD camera (Eagle CCD, FEI) at a magnification of 80,000×, a dose of ~20 electrons/Å^2^, and a defocus range of 1.5 ~ 3 μm in an FEI Tecnai F20 electron microscope operated at 200 kV.

### Gold labelling of the SMAs of the CeHSP17 protein on EM grids

The SMA sample (8 μl) was loaded on the EM grids. Excess liquid was removed by touching the edge of the grid with Waterman filtrate paper, the grid was then placed upside-down on a droplet of 5 nm Ni-NTA-Nanogold (Nanoprobes), incubated for 30 minutes at room temperature, the grid was then washed upside-down using a droplet of buffer containing 20 mM Tris (pH 7.6), 150 mM NaCl, 8 mM imidazole for 1 min. The grid was then rinsed six times consecutively in water before EM analysis.

### Single particle image processing and 3D reconstruction

The image processing software package EMAN2^47^, was used for the micrograph evaluation, particle picking, CTF correction, 2-D reference-free class averaging, initial model building and 3-D refinement. Symmetries of C6/D3/D6/TET of the spherical oligomer were validated one by one on the basis of the apparent 2-fold and 3-fold features in the 2-D reference-free averages. Briefly, a quick initial model was established with the specified symmetry imposed, then a quick refinement targeting at low resolution was performed before refining 3 more cycles without assuming any symmetry. The correct symmetry would be the one with which the map is preserved even without imposing symmetry while the incorrect symmetries would be the ones with which the maps are degraded rapidly during refinement. As a part of refinement, the gold standard resolution is one of the major changes in *e2refine_easy.py* of EMAN2.1. The resolution for the final map was estimated by the 0.143 criterion of FSC curve without any mask^48^. A 14.5 Å Gauss low-pass filter was applied to the final 3D map, and the Chimera^49^ software package was used to fit the coordinates of wheat HSP16.9 (PDB code 1GME) into the 3D map of CeHSP17 spherical oligomer, using one dimer of the HSP16.9, applying the tetrahedral symmetry.

### Tilt-pair validation

Tilt-pair validation data were collected at two goniometer angles, 0° and −10°, for each region of the grid. A particle set of 47 particles from two tilt pairs were used for the validation test (Supplementary Table S1). The test was performed using *e2tiltvalidate.py* program in EMAN2.07.

## Author Contributions

K.M.Z. and A.N.E. designed and performed most of the experiments. Z.W. and H.L.H. collected some cryo-EM data. X.D.S. and X.P.L. did some biochemical experiments. Z.W. and C.L. analysed some data. X.F., C.C.Y. and Z.C. designed and supervised the study and wrote the manuscript.

## Additional information

**Accession Codes:** The electron microscopy map of the CeHSP17 24-subunit oligomer has been deposited in the Electron Microscopy Data Bank with accession code EMDB2800.

## Supplementary Material

Supplementary Information Supplementary Information

Supplementary Information Supplementary Movie S1
