# Supplementary material for: A Novel Mechanism for Small Heat Shock Proteins to Function as Molecular Chaperones
Source: Sci Rep. 2015 Mar 6;5:8811. doi: 10.1038/srep08811 (PMC4351549; doi:10.1038/srep08811)
Supplement: Supplementary Information [file srep08811-s1.pdf]

Supplementary Information

A Novel Mechanism for Small Heat Shock Proteins to Function as Molecular Chaperones

Kaiming Zhang[1,*], Anastasia N. Ezemaduka[2,*], Zhao Wang[1,§*], Hongli Hu[1,†], Xiaodong Shi[2,¶], Chuang Liu[1,ξ], Xinping Lu[3], Xinmiao Fu[2], Zengyi Chang[2], Chang-Cheng Yin[1]

[1]Department of Biophysics, College of Basic Medical Sciences, Peking University Health Science Centre, Beijing 100191, China

[2]State Key Laboratory of Protein and Plant Gene Research, School of Life Sciences, Peking University, Beijing 100871, China

[3]School of Life Sciences, Tsinghua University, Beijing 100084, China

[4]Centre for Protein Science, Peking University, Beijing, 100871, China

[§] Present address: National Centre for Macromolecular Imaging, Verna and Marrs McLean Department of Biochemistry and Molecular Biology, Baylor College of Medicine, One Baylor Plaza, Houston, TX 77030, USA.

[†]Present address: Wadsworth Centre, New York State Department of Health, Albany, New York 12201, USA.

[¶]Present address: Jiangsu Province Key Laboratory of Anaesthesiology, Xuzhou Medical College, Xuzhou, 221002, China

[ξ] Present address: Department of Structural Biology, School of Medicine, University of Pittsburgh, Pittsburgh, PA 15260, USA

*These authors contributed equally to this work. Correspondence and requests for materials should be addressed to C.C.Y. (ccyin@hsc.pku.edu.cn) or Z.C. (changzy@pku.edu.cn)

Supplementary Figure S1: *In vitro* chaperone activity analysis of CeHSP17 spherical oligomers. (a) Light scattering analysis of the aggregation of insulin (0.25 mg/ml) as induced by 20 mM TCEP at 50 °C in the absence or presence of CeHSP17 (0.25 mg/ml). **(b)** Light scattering analysis of the heat induced aggregation of ADH (1 mg/ml) at 50 °C in the absence or presence of CeHSP17 (0.25 mg/ml). **(c)** Light scattering analysis of the CeHSP17 spherical oligomers (0.25 mg/ml) incubated at 50 °C. **(d)** Size-exclusive chromatography analysis of the purified CeHSP17 protein (1 mg/ml) that was untreated, pre-treated at 50 °C for 30 min, or pre-treated at 50 °C for 30 min and then incubated at 4 °C overnight before analysis. All samples were centrifuged before the supernatant was loaded into the column. **(e)** A representative image for the sample that was cooled down to 4 °C after being heated at 50 °C. The scale bar represents 50 nm.

Supplementary Figure S2: The non-SMA oligomers lack chaperone activity. (a) Light scattering analysis of the aggregation of insulin (0.25 mg/ml) as induced by 20 mM TCEP at 50 °C in the absence or presence of CeHSP17 SMAs (0.25 or 0.5 mg/ml). **(b)** A representative image for the supernatant sample. The scale bar represents 50 nm. **(c)** A representative image for the pellet sample. The scale bar represents 200 nm. **(d)** Light scattering analysis of the heat induced aggregation of ADH (1 mg/ml) at 50 °C in the absence or presence of non-SMA CeHSP17 oligomers. **(e)** Light scattering analysis of the aggregation of insulin (0.25 mg/ml) as induced by 20 mM TCEP at 50 °C in the absence or presence of non-SMA CeHSP17 oligomers. The SMAs were removed as pellet from the heat-shocked (at 50 °C) CeHSP17 sample by centrifugation at 15,000 g for 10 min, and the supernatant containing non-SMA oligomers was subjected to chaperone activity assay. Both heat-induced aggregation of ADH (panel **d**) and TCEP-induced aggregation of insulin (panel **e**) could not be suppressed by non-SMA oligomers.

On the contrary, non-SMA oligomers promote the aggregation of ADH and insulin, presumably by forming co-aggregates with non-native proteins. The light scattering readout at the zero time point was set as zero upon adding the substrates.

Supplementary Figure S3 (related to Figure 5): Three-dimensional reconstruction and tilt-pair validation of CeHSP17 spherical oligomers. **(a)** Representative field of EM image of the CeHSP17 spherical oligomers. The samples were imaged using an FEI F20 electron cryo-microscope operated at 200 kV and at liquid nitrogen temperature. Images were acquired on a 4k × 4k CCD camera at 62,000 nominal magnification of microscope using low dose mode (~20 electron/$Å^2$) and a defocus range of 2~4 μm. The scale bar represents 50 nm. **(b)** Representative reference free 2D class averages. **(c)** Representative class averages and corresponding projections of 3D reconstruction map from raw particle images. The scale bar represents 10 nm. **(d)** The Euler angle distribution of the particle images used for 3D reconstruction. **(e)** Gold standard FSC plot for the 3D reconstruction. **(f)** Result of tilt-pair validation. The red circle denotes particle pair that cluster around the experimental tilt geometry.

Supplementary Figure S4: Three-dimensional structure model of the CeHSP17 hexamers that are proposed to be the structural units of the CeHSP17 spherical oligomers. **(a)** Top view. **(b)** Side view. **(c)** Inside to outside view. In each panel, the upper part represents the EM density map and the lower part represents the structure model obtained by rigid body docking using the determined crystal structure of the homologous wheat HSP16.9 dimer (PDB code 1GME).

Supplementary Table S1 (related to Supplementary Figure S3): Tilt geometry computed for tilt pair of images, using the final 3D model of CeHSP17 oligomer.

	CCD Image pair
Total particle pairs	47
Particle Pairs in Cluster	18
Fraction in cluster	38%
Mean Tilt Angle	12.88
RMSD Tilt Angle	5.12
Mean Tilt Axis	-79.58
RMSD Tilt Axis	30.29
Tilt Angle via microscope	10

Supplementary Movie S1 (related to Fig. 5 and Supplementary Fig. S4): Showing different views along symmetry axes, one "trimer of dimers" of EM density map and crystal structure docking using homologue protein (wheat HSP16.9 dimer (PDB code 1GME)).

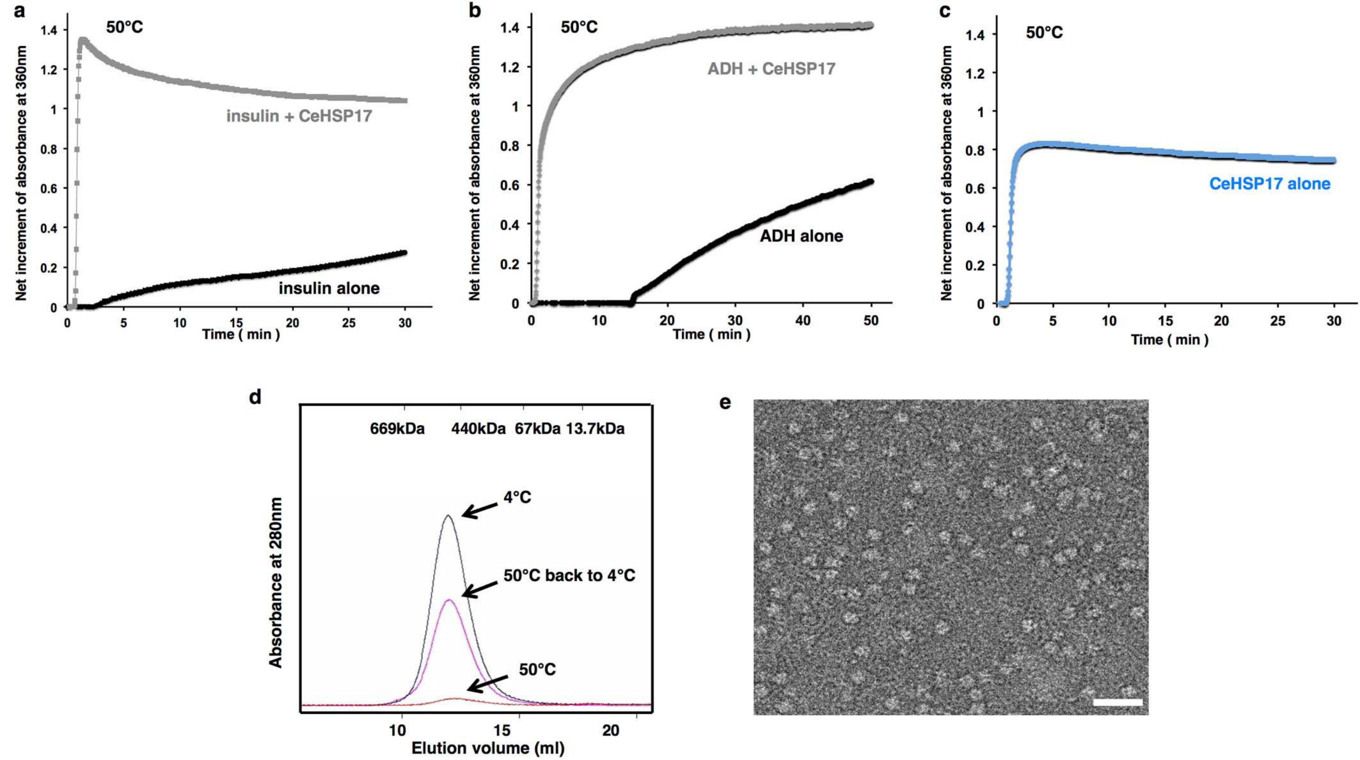

a
50°C
insulin + CeHSP17
insulin alone
Net increment of absorbance at 360nm
Time (min)
b
50°C
ADH + CeHSP17
ADH alone
Net increment of absorbance at 360nm
Time (min)
c
50°C
CeHSP17 alone
Net increment of absorbance at 360nm
Time (min)
d
669kDa
440kDa
67kDa
13.7kDa
4°C
50°C back to 4°C
50°C
Absorbance at 280nm
Elution volume (ml)
e

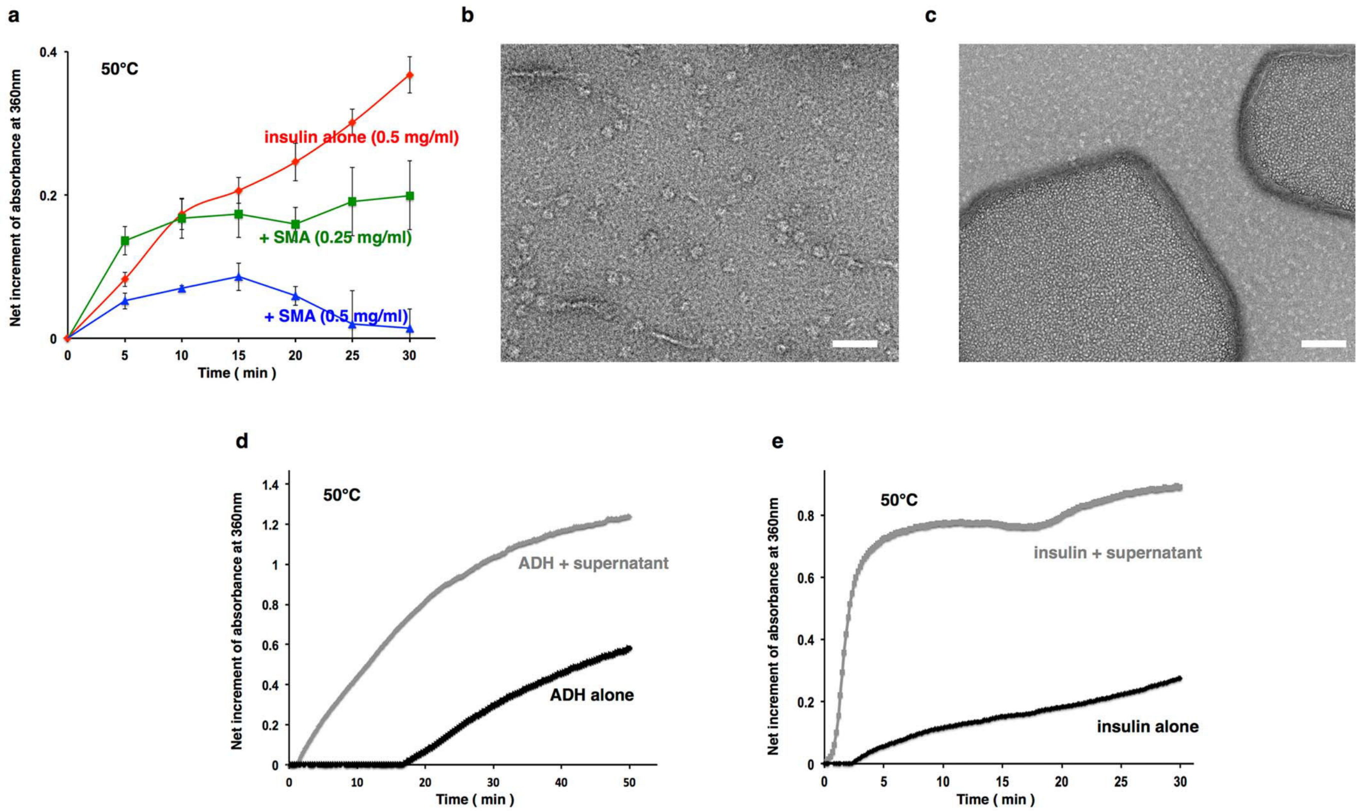
a
b
c
d
e
50°C
Net increment of absorbance at 360nm
insulin alone (0.5 mg/ml)
+ SMA (0.25 mg/ml)
+ SMA (0.5 mg/ml)
Time (min)
0
0.2
0.4
5
10
15
20
25
30
ADH + supernatant
ADH alone
0.6
0.8
1
1.2
1.4
40
50
insulin + supernatant
insulin alone

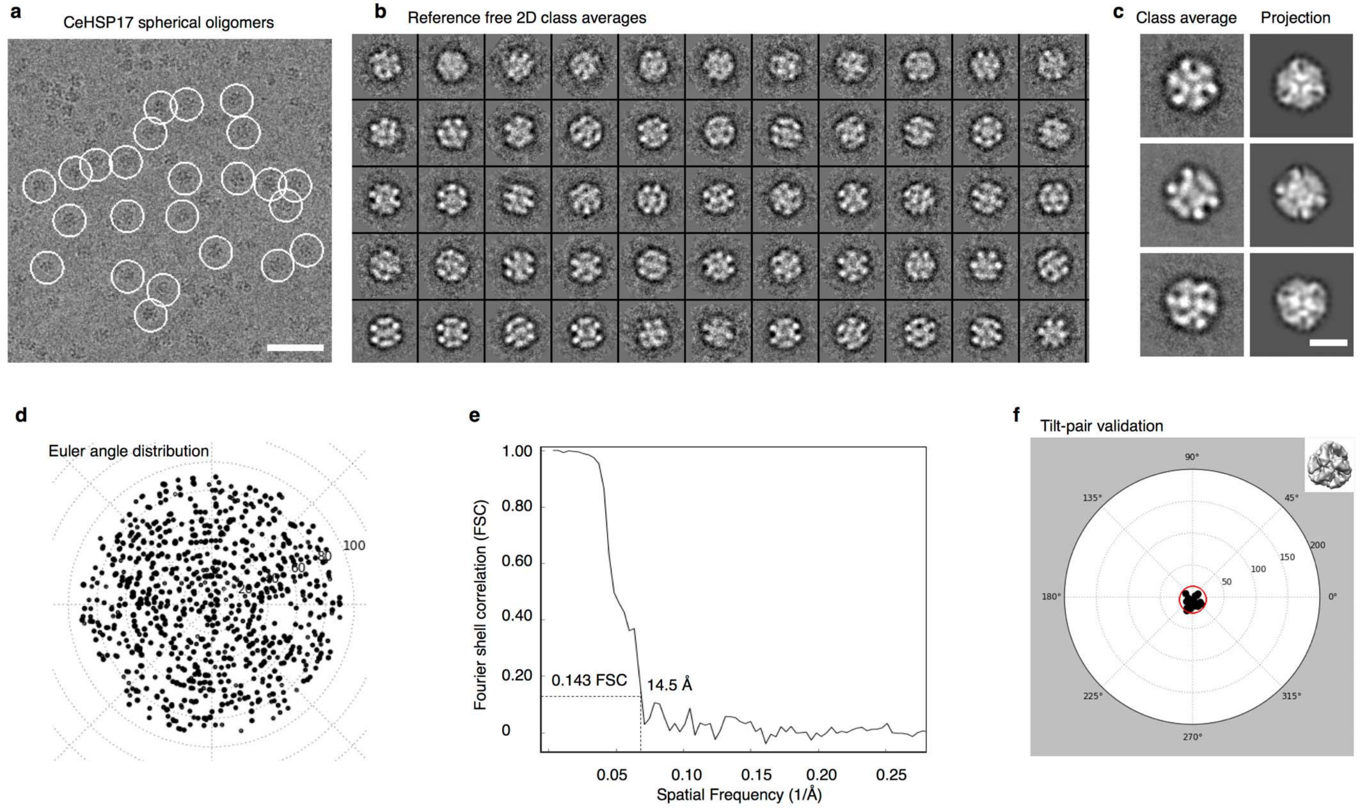
a
CeHSP17 spherical oligomers
b
Reference free 2D class averages
c
Class average
Projection
d
Euler angle distribution
20
40
60
80
100
e
Fourier shell correlation (FSC)
1.00
0.80
0.60
0.40
0.20
0
0.143 FSC
14.5 Å
0.05
0.10
0.15
0.20
0.25
Spatial Frequency (1/Å)
f
Tilt-pair validation
90°
135°
45°
180°
0°
225°
315°
270°
50
100
150
200

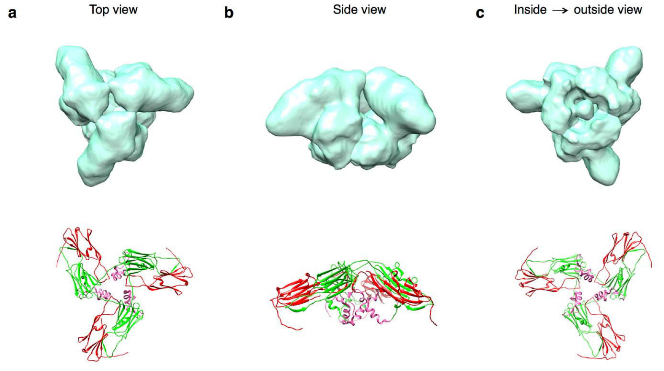
a
Top view
b
Side view
c
Inside → outside view
